# Replication of Human Norovirus in Mice after Antibiotic-Mediated Intestinal Bacteria Depletion

**DOI:** 10.3390/ijms231810643

**Published:** 2022-09-13

**Authors:** Cristina Santiso-Bellón, Roberto Gozalbo-Rovira, Javier Buesa, Antonio Rubio-del-Campo, Nazaret Peña-Gil, Noemi Navarro-Lleó, Roberto Cárcamo-Calvo, María J. Yebra, Vicente Monedero, Jesús Rodríguez-Díaz

**Affiliations:** 1 Department of Microbiology, School of Medicine, University of Valencia, Av. Blasco Ibáñez 17, 46010 Valencia, Spain; 2 Instituto de Investigación INCLIVA, Hospital Clínico Universitario de Valencia, 46010 Valencia, Spain; 3 Department of Food Biotechnology, Instituto de Agroquímica y Tecnología de Alimentos (IATA-CSIC), Av. Agustín Escardino 7, 46980 Paterna, Spain

**Keywords:** norovirus, antibiotic, microbiota, mice, virus shedding

## Abstract

Human noroviruses (HuNoVs) are the main cause of acute gastroenteritis causing more than 50,000 deaths per year. Recent evidence shows that the gut microbiota plays a key role in enteric virus infectivity. In this context, we tested whether microbiota depletion or microbiota replacement with that of human individuals susceptible to HuNoVs infection could favor viral replication in mice. Four groups of mice (n = 5) were used, including a control group and three groups that were treated with antibiotics to eliminate the autochthonous intestinal microbiota. Two of the antibiotic-treated groups received fecal microbiota transplantation from a pool of feces from infants (age 1–3 months) or an auto-transplantation with mouse feces that obtained prior antibiotic treatment. The inoculation of the different mouse groups with a HuNoVs strain (GII.4 Sydney [P16] genotype) showed that the virus replicated more efficiently in animals only treated with antibiotics but not subject to microbiota transplantation. Viral replication in animals receiving fecal microbiota from newborn infants was intermediate, whereas virus excretion in feces from auto-transplanted mice was as low as in the control mice. The analysis of the fecal microbiota by 16S rDNA NGS showed deep variations in the composition in the different mice groups. Furthermore, differences were observed in the gene expression of relevant immunological mediators, such as IL4, CXCL15, IL13, TNFα and TLR2, at the small intestine. Our results suggest that microbiota depletion eliminates bacteria that restrict HuNoVs infectivity and that the mechanism(s) could involve immune mediators.

## 1. Introduction

Acute gastroenteritis (AGE) is one of the 10 more common causes of death worldwide. Furthermore, the World Health Organization (WHO) considers that AGE is the second most common etiology of death in developing countries [1]. AGE caused by enteric viruses is the most frequent type of diarrheal disease, with human noroviruses (HuNoVs) being one of the most relevant causal agents. The infection routes for HuNoVs are usually the consumption of contaminated food or water, person-to-person transmission via direct contact, exposure to aerosols, and the fecal–oral route [2].

The genus *Norovirus* belongs to the *Caliciviridae* family. These viruses possess a single stranded, positive sense RNA genome of 7.7 kb that encodes three open reading frames (ORF). ORF1 codes for a polyprotein that is proteolyzed into seven non-structural mature proteins (NS1 to NS7). These non-structural proteins include the viral polymerase required for viral replication. ORF2 encodes the capsid protein, known as viral protein 1 (VP1), while ORF3 codes for a minor capsid structural protein VP2 [3].

NoVs have been classified into 10 genogroups (GI-GX) based on the phylogeny of the VP1 amino acid sequences [4]. Genogroups GI, GII and GIV are able to infect humans. Among them, GI and GII are found in most cases. Genogroups are further divided into genotypes, with GII.4 being the most frequent cause of NoV outbreaks [4,5].

The intestinal microbiota has emerged as a key factor in enteric virus infections [6,7]. It was determined that the gut bacteria are necessary for the persistence of enteric murine NoVs in mice [8] and, in a similar way, it was shown that HuNoVs can replicate in human B lymphocytes when the fecal samples used as source of HuNoVs are unfiltered (not devoid of accompanying bacteria), or in the presence of enteric bacteria such as *Enterobacter cloacae* [9]. This bacterium expresses histo-blood group antigen-like molecules on its surface, allowing the attachment of HuNoVs to target cells [10]. Gnotobiotic pigs in which human intestinal microbiota was transplanted showed stimulated HuNoVs (GII.4/2006b strain) infection [11] and there are also evidences that the gut microbiota might have an influence in HuNoV infections in humans [12]. On the other hand, recent results showed that the mouse gut microbiota may restrict infection in mice of another enteric virus causing AGE: rotavirus (RV) [13,14]. These opposing results still depict an unclear role of the microbiota in the infection of AGE-causing viruses. Interestingly, HuNoVs do not replicate in immunocompetent mice, but they can replicate in BALB/c Rag-γc-deficient mice [15].

In this context, we decided to test the effects of microbiota eradication and/or its replacement with human intestinal microbiota on the ability of HuNoVs to infect mice. We showed that HuNoVs (from the GII.4 Sydney [P16] genotype) replicated in immunocompetent mice with altered microbiota. This observation supports a protective role of the microbiota in this experimental model and provides a new small animal model where associations between the gut microbiota, the immune system and the level of replication of HuNoVs could be studied.

## 2. Materials and Methods

### 2.1. HuNoV Stock Preparation

A stool sample containing HuNoVs belonging to the GII.4 Sydney [P16] genotype was suspended at 10% in PBS. The viral suspension was centrifuged at 15,000× *g* to remove bacteria and debris. The clarified supernatant was used to pellet the virus at 150,000× *g* in a SW41 rotor coupled to a Beckman L80 ultracentrifuge for two hours. The pellet containing the virus was resuspended in 10 mL of PBS. After preparation, HuNoV stock was titrated by qPCR as previously described [16].

### 2.2. Human Fecal Transplant Preparation

The collection and preservation of fecal samples from four healthy newborns between one and three months of age was previously described [17]. These fecal samples, resuspended in 2X-concentrated brain–heart infusion (Pronadisa) with 0.1% cysteine and 40 g/L skim milk (Scharlab), pooled and stored at −80 °C, were used in this study. Fecal pellets from each group of mice were collected before the experiments and preserved in the same medium for performing the self-transplantation.

### 2.3. Experimental Procedures with Mice

The experimental procedures with mice were carried out basically as previously described [14]. Briefly, four groups of five C57BL/6J female 6-week-old mice were used. Each group of animals was kept in individual cages (5 animals per cage). Three groups were treated with an antibiotic cocktail (1 g/L ampicillin, 1 g/L metronidazole, 1 g/L neomycin and 0.5 g/L vancomycin) in drinking water *ad libitum* for three weeks changing the solution every three days. One week before fecal transplantation, the standard rodent diet was substituted by purified-defined germ-free diet (AIN-93G, Envigo). The control group was not treated with antibiotics. Twenty-four hours after antibiotic treatment, two groups of mice were subject to fecal material transplantation (FMT). One group of mice (self-FMT) was transplanted with the preserved microbiota from the same group of mice before antibiotic treatment as previously described [14]. A second group of mice (hFMT) received a FMT using the same procedure with a pool of bacteria from infant feces.

Six days after FMT the mice were orally inoculated with 1.5 × 10^7^ genome copy equivalent (GCE) of HuNoV in 100 μL of PBS. After HuNoV dosing, the antibiotic-treated group without FMT continued the antibiotic treatment, whereas one day before FMT, antibiotics were omitted in the FMT groups. Mice fecal pellets were collected daily for 7 days and kept at −20 °C. The mice were euthanized at 7 days post-infection (dpi) and the small intestine was removed and stored in RNAlater (Sigma) at −80 °C for further analysis. An outline of the animal experimental procedure is depicted in Supplementary Figure S1.

### 2.4. Quantification of HuNoVs from Stool Samples by RT-qPCR

The viral RNA was extracted from mice fecal samples using the NucleoSpin RNA Virus (Macherey-Nagel) kit following the manufacturer’s instructions. The primers and probe sequences utilized for RT-qPCR have previously been described [18]. The amplification of RNA samples was performed with one-step TaqMan RT-qPCR using the RNA UltraSense One-Step quantitative system (Thermo Fisher Scientific, Waltham, MA, USA). The standard curve was generated by serial end-point dilution, amplifying 10-fold dilutions of the quantified plasmid containing the HuNoV target sequence by RT-qPCR in triplicates. The plasmid utilized to quantify the viral load was custom synthesized by GenArt (Thermo Fisher Scientific) containing the NoV sequence previously described [18].

### 2.5. Quantification of Cytokine and Glycosyltransferase mRNA Expression Level

The expression levels of genes encoding cytokines IL1β, IL4, IL6, CXCL15, IL10, IL12, IL13, TNFα, IFNγ and TLR2 as well as the fucosyl transferase FUT2 was studied as previously described [14]. Briefly, mRNA was extracted from 100 mg of small intestine using Trizol (Thermo Fisher Scientific, Waltham, MA, USA). The RNA was retro-transcribed using the SuperScript III enzyme (Thermo Fisher Scientific) and random primers following the manufacturer’s instructions. The expression level of GAPDH, RPLPO and HPRT housekeeping genes was used as reference. The quantitative PCRs were performed in a LightCycler480 Instrument SW1.5 (Roche Life Science, Basel, Switzerland)) and in the StepOnePlus Real-Time PCR System (Applied Biosystems part of Thermo Fisher, Waltham, MA, USA) [14]. The expression analysis was performed with Rest Software [19].

### 2.6. Microbiota Profiling in Mice Stool Samples

Total rDNA 16S in mice feces was quantified by qPCR as previously described [20]. Intestinal microbiota composition analysis in mice was performed by isolating total bacterial DNA from fecal pellets as previously described [14]. Bar-coded amplicons of the 16S rDNA V3–V4 region were then subject to 2 × 300 bp paired-end run in a MiSeq-Illumina platform (SCSIE, University of Valencia, Valencia, Spain). After data demultiplexing and filtering using the Illumina bcl2fastq program, reads were checked for quality, adapter trimmed and filtered with AfterQC and FastQC v0.11.8 (http://www.bioinformatics.babraham.ac.uk, accessed on 15 February 2022) tools. Sequencing data were analyzed with QIIME software V1.9.1 [21]. Chimeric sequences were removed using the USEARCH 6.1 algorithm. Reads were clustered into operational taxonomic units (OTUs) based on a 97% identity threshold. Sequences were aligned to the Greengenes core reference database (version 13.8) using PyNAST and assigned taxonomically with UCLUST. A total of 5,374,548 reads were obtained, with a mean of 268,727 sequences per sample. After rarefying to the minimum library size (133,859 reads), data normalized by total-sum scaling were analyzed with MicrobiomeAnalyst pipeline (https://www.microbiomeanalyst.ca/; [22], accessed on 18 February 2022).

## 3. Results

### 3.1. HuNoVs Efficiently Replicate in Mice with Depleted Microbiota

**Figure 1:**
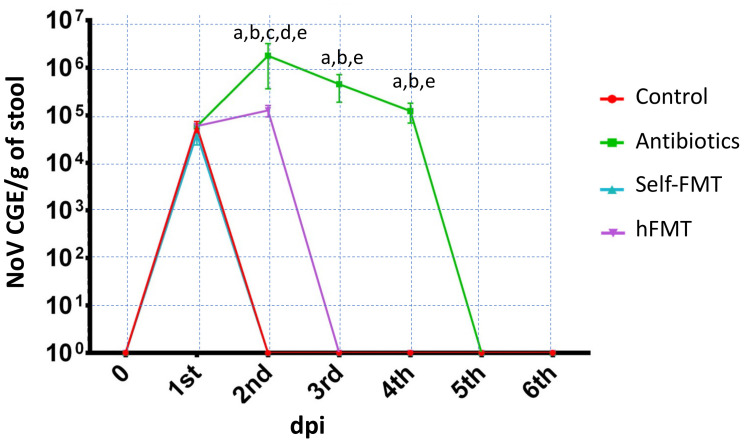
**HuNoV GII.4 Sydney [P16] shedding in mouse stools**. GCE/g of stool are shown for the control mice, Ab-treated mice and mice subject to hFMT and self-FMT, respectively. Mouse feces were sampled until dpi 6. The letters indicate statistically significant differences (*p* < 0.05) in the GCE between the groups (a, Ab vs. control; b, Ab vs. self-FMT; c, hFMT vs. control; d, hFMT vs. self-FMT; e Ab vs. hFMT). The error bars are standard deviations (n = 5). The limit of detection was 10^4^ GCE/g of stool. Ab, antibiotic; hFMT, human fecal microbiota transplantation; self-FMT, self-fecal microbiota transplantation; dpi, days post infection.

Prior experiments from our group in mice with another enteric virus (RV) showed that antibiotic treatment enhanced the replication of human RV in the murine model [14]. In the present experiments, we wanted to study whether a similar situation could be observed for HuNoVs and which was the impact of FMT performed with human fecal microbiota. Similar to data reported for RV, the group treated with antibiotics (Ab group) supported the replication of HuNoVs for four days (Figure 1). The virus could be detected in the feces of control animals with a peak at one day post-infection (dpi; 5.8 × 10^4^ genome copy equivalent (GCE)/g feces), which rapidly dropped, with HuNoV being under the detection limit at dpi 2 (Figure 1). Contrarily, compared to the control animals, at dpi 2, viral shedding in Ab-treated mice and in mice subject to human FMT (hFMT) was 31.03- and 2.24-fold higher (1.8 × 10^6^ GCE/g and 1.3 × 10^5^ GCE/g, respectively). The lower levels of HuNoVs detected in the control animals and their faster clearance were indicative of an inefficient replication, in contrast to the prolonged viral shedding in Ab-treated mice and in the hFMT group. In mice receiving self-FMT, a lack of HuNoV replication was observed, and the virus was detected in mice feces at similar levels compared to control mice at dpi 1, with the absence of viral shedding at dpi 2 (Figure 1).

### 3.2. Impact of Fecal Microbiota Transplant on Mouse Microbiota

**Figure 2:**
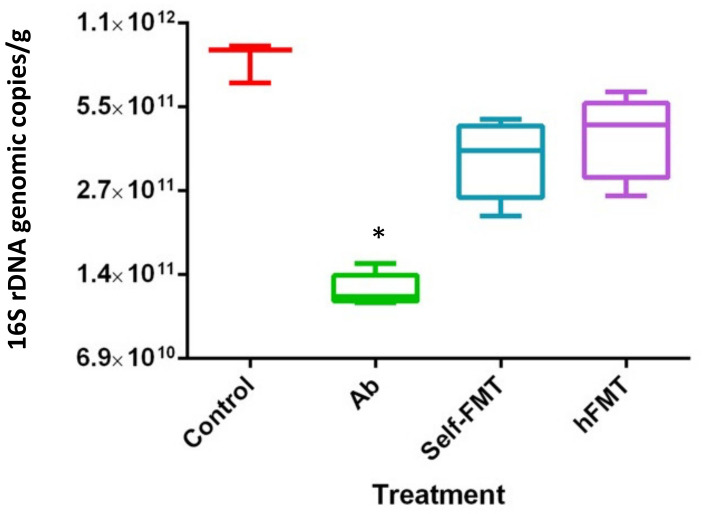
**Total bacterial 16S rDNA quantification by qPCR**. The 16S rDNA genomic copies/g of stools are presented for the control mice, Ab-treated mice and mice subject to self-FMT and hFMT, respectively. Ab-treated mice had a significantly lower amount of 16S rDNA copies than the other three groups (*p* < 0.05), indicated by an asterisk (*). Ab, antibiotic; hFMT, human fecal microbiota transplantation; self-FMT, self-fecal microbiota transplantation.

The results of viral shedding indicate that the Ab treatment was the responsible for the permissiveness to HuNoV infection in mice, pointing to the microbiota composition and abundance as a key player in HuNoV infectivity in this animal model. After the Ab treatment, the number of bacteria, calculated as the number of copies of 16S rDNA in stools, was drastically reduced (Figure 2). Mice subject to one or the other FMT treatment showed an increase in the number of bacteria, although the original numbers present in the control animals were not reached (Figure 2).

**Figure 3:**
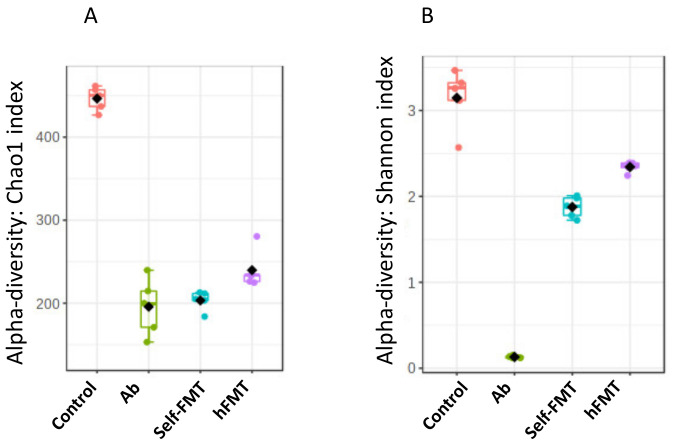
**Changes in intestinal bacterial richness and diversity**. Microbial α-diversity. (**A**) Microbial richness (Chao1) and (**B**) diversity (Shannon) indexes are shown. Ab, antibiotic; hFMT, human fecal microbiota transplantation; self-FMT, self-fecal microbiota transplantation.

**Figure 4:**
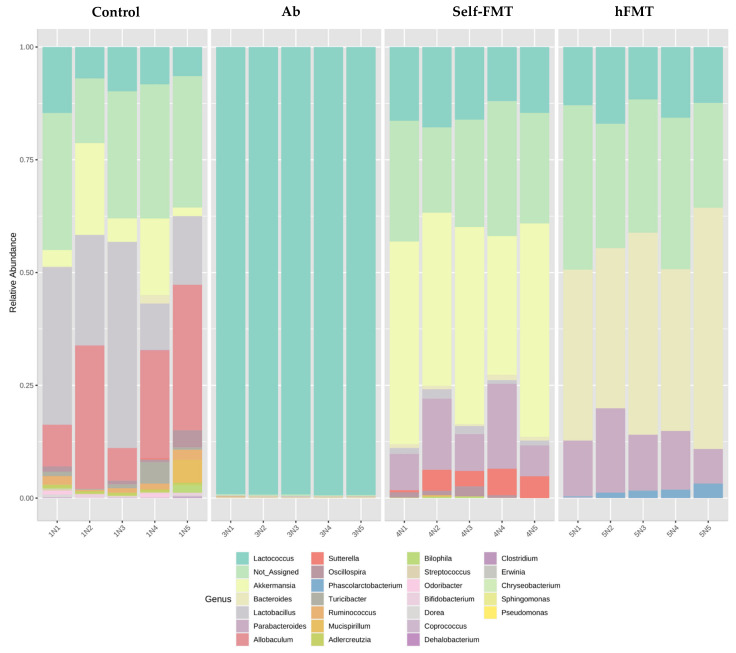
**Changes in intestinal bacterial composition**. Relative abundance of bacterial taxa (genus levels) found in the feces of the mice (n = 5) from the different treatment groups. Ab, antibiotic; hFMT, human fecal microbiota transplantation; self-FMT, self-fecal microbiota transplantation.

**Figure 5:**
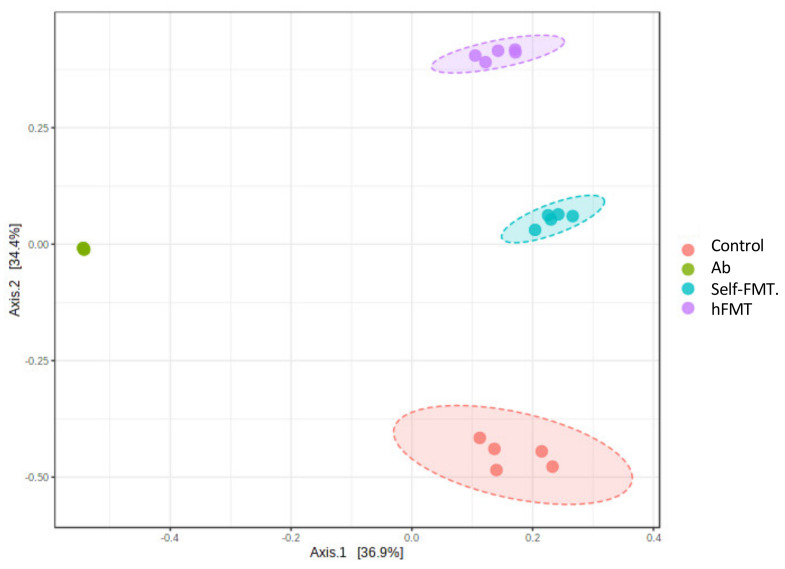
**Differences in microbial global composition after the different treatments**. A principal coordinate analysis (PCoA) of the Bray–Curtis dissimilarity indexes of samples based on OTUs is shown (PERMANOVA F–value = 53.642; R^2^ = 0.90957; *p* < 0.001). Ab, antibiotic; hFMT, human fecal microbiota transplantation; self–FMT, self-fecal microbiota transplantation.

The fecal microbiota composition in the animals before the HuNoV challenge was determined by 16S rDNA NGS, showing that bacterial richness and diversity was severely affected by the treatments (Figure 3). Chao1 richness index was reduced in all groups compared to the control (*p* = 7.2942 × 10^−13^; F-value = 198.34, ANOVA) but was higher in the hFMT animals. Diversity (Shannon diversity index) was also lower (*p* = 2.3244 × 10^−13^; F-value = 229.68, ANOVA), but was higher in the hFMT group compared to self-FMT group, indicating uneven microbial distribution after treatments. As already reported in similar experimental setting [14], the three distinct treatments caused profound remodeling of the microbiotas and all groups could be distinguished by their microbial profiles in the feces (Figure 4). In the Ab group, members of the genus *Lactococcus* accounted for most of the microbiota (Figure 4). Furthermore, β-diversity analysis clearly differentiated the distinct treatment groups (Figure 5).

**Figure 6:**
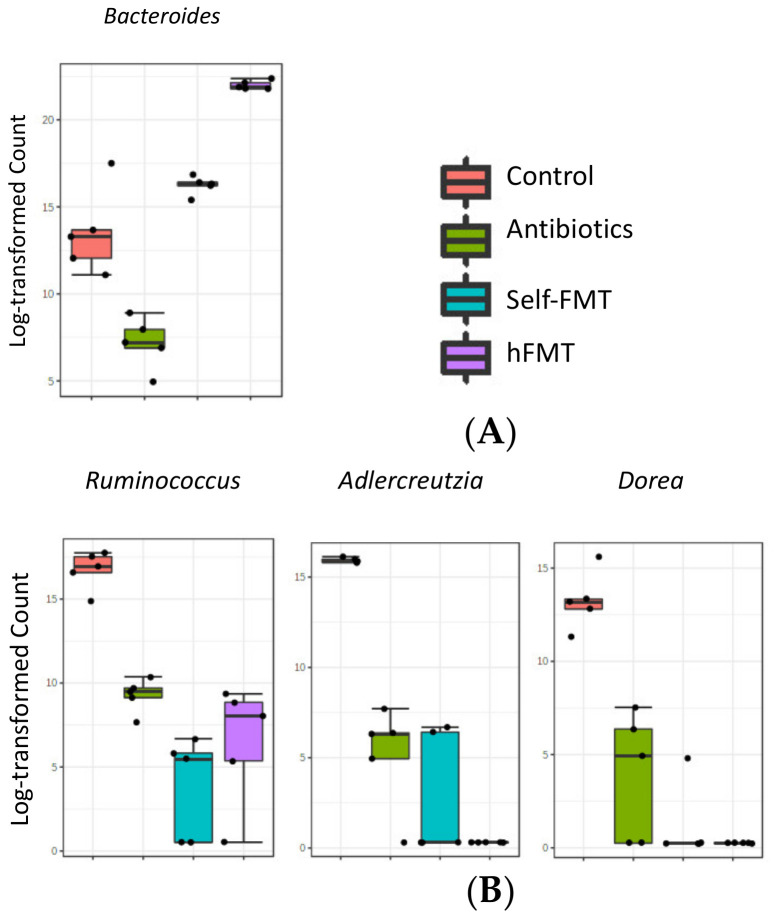

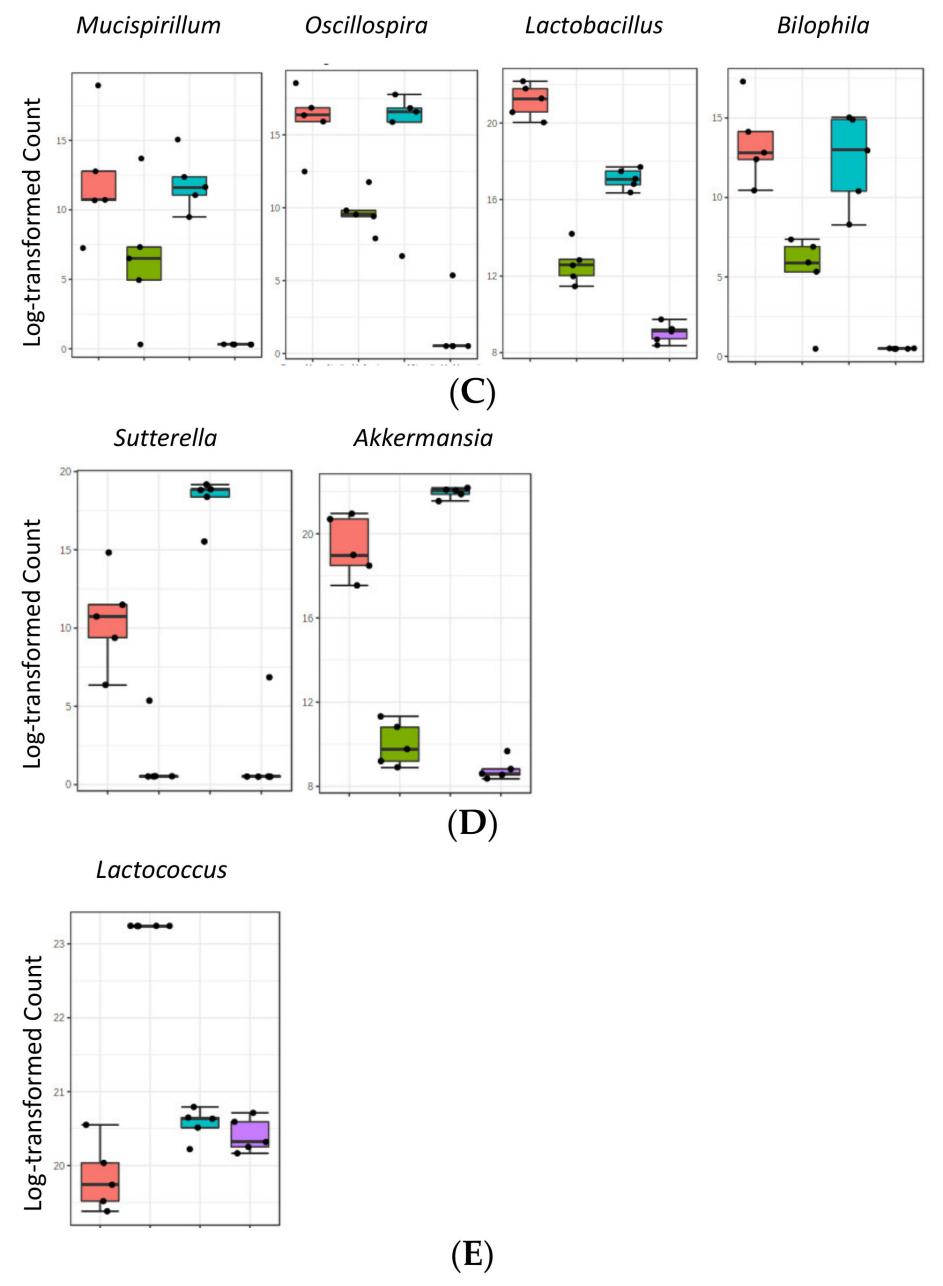
**Bacterial genera showing differences between treatment groups**. LEfSe analysis [23] was performed to detect fecal bacterial taxa that differed in abundance in a particular treatment. The different box-plots represent abundance (log transformation of data normalized by total-sum scaling × 10^7^) of bacterial genera with LDA scores > 4 when all treatment groups were analyzed. (**A**) Bacterial genera increased in hFMT; (**B**) Genera ablated or lowered by Ab treatment; (**C**) Genera partially restored by self-FMT; (**D**) Genera increased after self-FMT; (**E**). Genera increased in Ab-group. Red, control animals; green, Ab-treated animals; blue, Self-FMT; magenta, hFMT. Ab, antibiotic; hFMT, human fecal microbiota transplantation; self-FMT, self-fecal microbiota transplantation.

As in previous research [14], the microbiota present in the pooled infant feces was not totally engrafted in mice after hFMT (data not shown), but these mice were characterized by elevated numbers of *Bacteroides*, which were low in the control mice (Figure 6A). Genera such as *Adlercreutzia*, *Ruminococcus* and *Dorea* were depleted after the Ab treatment and they did not recover after the FMT treatments (Figure 6B). The evident differences in α and β diversities showed that a completed restoration of mice microbiota was not achieved by means of self-FMT. However, the levels of bacteria from some genera (e.g., *Oscillospira*, *Lactobacillus*, *Mucispirillum* or *Bilophila*) were partially restored in mice via this treatment (Figure 6C). Self-FMT also produced elevated proportions of *Akkermansia* and *Sutterella* when compared to the control mice (Figure 6D). As already mentioned, the more abundant taxon after antibiotic treatment was *Lactococcus* (Figure 6E). Similar to our previous study with RV and Ab-treated mice [14], low microbial diversity, together with the eradication of representative taxa of the intestinal microbiota, was linked to the permissiveness of HuNoV replication in mice.

### 3.3. Downregulation of TNFα Gene Expression in the Gut Associates with HuNoV Infection in Mice

**Figure 7:**
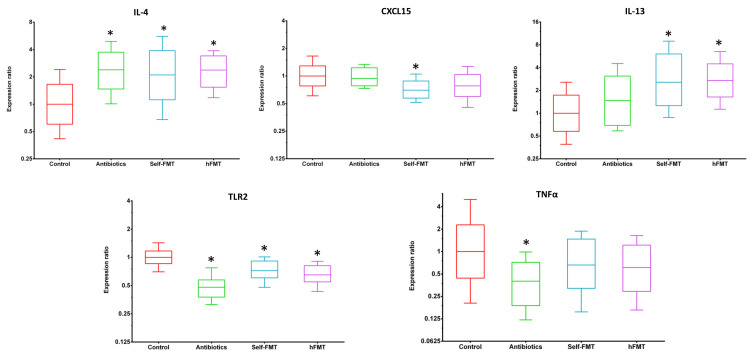
**Differences in gene expression in the small intestine of infected mice**. Expression of immune system mediators IL4, CXCL15, IL13, TNFα and TLR2 determined by RT-qPCR in mice from the different groups at 7 dpi (n = 5) are shown. The level of significance is indicated relative to the control mice (*p* < 0.05) is indicated by an asterisk (*).

The gene expression levels of a panel of cytokines and other innate immune system mediators, including IL1β, IL4, IL6, CXCL15, IL10, IL12, IL13, IFNγ, TNFα and TLR2, were studied by RT-qPCR in the small intestine of mice at 7 dpi. IL1β, IL6, IL10, IL12n and IFNγ expression levels did not differ between the animal groups. The expression of IL4 was upregulated in all antibiotic treated groups, as well as IL13 that was upregulated in both groups that received an FMT (Figure 7), whereas a downregulation effect was generally observed in the remaining tested genes. CXCL15 messenger levels were lower in the self-FMT group. Similarly, the expression level of TLR2 was diminished in all groups treated with Ab. More interestingly, the expression level of TNFα was reduced exclusively in the group where HuNoV replicated more efficiently. The expression of the FUT2 gene, whose product is involved in α1,2-fucosylation during the synthesis of mucosal H type-1 antigen (fucosyl-α1,2-galactopyranosyl-β1,3-N-acetyl-glucopyranoside), one of the HuNoV adhesin receptors, did not change in any treatment.

## 4. Discussion

The study of the biology of HuNoVs has been hampered by the lack of suitable cell culture models [24] and small animal models. In the last decade, this problem has been partially solved by the use of human intestinal enteroids [25], immunocompromised mice [15] and the utilization of the zebrafish model [26]. The results presented in this paper provide a new immunocompetent mouse model that allows the study of HuNoVs–host interactions, including the role of the gut microbiota and the immune system mediators.

It has been largely discussed whether the gut microbiota acts by limiting or potentiating NoV replication [6,27]. Until the 2010s, it was generally accepted that a healthy gut microbiota should protect against viral enteric pathogens [28]. However, in the 2010s, several original works pointed to the opposite direction, suggesting an active role of the gut microbiota in enteric virus infections such as NoV, RV, poliovirus and reovirus [9,29,30,31]. Nevertheless, recent research also pointed to the gut microbiota as a restrictor of enteric virus infections [14,32,33]. The results presented in this paper with HuNoVs correlate with experiments from our group with human RV, such as the depletion of certain bacterial groups by means of antibiotics allowing viral replication [14]. Furthermore, an increase in viral replication was absent when the mice were transplanted with human microbiota. This indicated that, in this model of FMT, no bacterial taxa promoting HuNoV replication were transferred to mice. Nevertheless, we detected a certain level of replication in the animals that received the hFMT, suggesting that the engrafted human microbiota was less restrictive to HuNoV infection compared to mouse native intestinal bacteria. These results differ from those obtained when we used human RV in the same mice model [14]. In this case, hFMT resulted in RV shedding in feces similar to animals that only received antibiotics. This suggests that the restriction that the microbiota causes on RV is weaker compared to HuNoVs. In the present experimental design, the negative effects on enteric virus infectivity attributed to the use of antibiotics but independent of microbiota changes [34] can be excluded. Indeed, self-FMT after antibiotic exposure completely restored the exclusion of HuNoVs in the mice. This restoration was more pronounced for HuNoVs compared to human RV [14], reinforcing again the idea that the viral exclusion promoted by the microbiota is more important in HuNoVs compared to human RV. However, it has to be pointed out that human RV replication in the mice, measured as viral shedding in feces, was much more efficient compared to HuNoVs.

The changes in the fecal microbiota composition presented in this paper paralleled those previously obtained when we studied human RV infection in mice [14], as the same mice strain, antibiotic dosage and mice diets were used, and the observed variations were reproduced. Thus, the fecal microbiota analyses allowed us to identify certain bacterial taxa that were augmented or decreased in the different experimental groups. From all that information, four bacterial genera were ablated or diminished after antibiotic treatment and partially recovered after self-FMT (*Oscillospira*, *Lactobacillus*, *Mucispirillum* and *Bilophila*), which restored the inability of HuNoVs to develop in the mouse. This suggests that, similar to RV [14], these bacteria might play a role in the restriction of HuNoV infection in mice. Owing to their probiotic characteristics, lactobacilli have been tested for their anti-NoV effects in several infection models. As examples, *Lacticaseibacillus rhamnosus* GG protected gnotobiotic pigs from HuNoVs (GII.4/2006b strain)-induced diarrhea and decreased viral shedding [35] and other probiotic bacteria were characterized as being able to interact with HuNoV P-particles and virions [35,36]. Other evidence exists on the participation of specific intestinal bacteria in viral restriction. As an example, *Blautia coccoides* was able to protect mice from encephalomyocarditis virus infection [37], *Candidatus* Arthromitus was linked to inhibition of murine RV [32] and *Clostridium* species have been shown to affect murine NoV replication [38]. It will be interesting to test which are the effects of supplementing these specific bacteria on HuNoVs and human RV infectivity in mice with antibiotic-induced microbiota depletion.

Other bacterial genus identified by the microbiota analysis as ablated by antibiotic treatment, *Ruminococcus*, emerges as a bacterial taxon with the potential of antiviral activity. These bacteria were negatively correlated with both RV and NoV susceptibility in humans [12]. They were also ablated in mice where human RV replicated more efficiently [14], they have been shown to interact with human RV ex vivo and they were able to partially block human RV infection in vitro [39]. The results obtained in this paper do not allow to identify any bacterial group that could facilitate HuNoV replication in the mouse model.

In addition to the direct effects that some specific bacteria might have on viral stability and viability, directly affecting the viral infectivity [31], it is well established that the gut microbiota is a strong modulator of the immune response. It is known that intestinal bacteria are necessary to mount the type I IFN response that controls viral infections [40]. Recent studies in mice also showed that intestinal bile acids metabolism carried out by *Clostridium scindens* triggers IFNλ response in the proximal small intestine, which was necessary to limit murine NoV replication [38]. Our results suggests that an anti-inflammatory environment triggered by antibiotic treatment (the anti-inflammatory cytokine IL4 was augmented, while the pro-inflammatory cytokine TNFα was significantly reduced) may play a role in the permissiveness to HuNoV replication in mice with altered microbiota. This situation was similar to that found for human RV in antibiotic-treated animals [14], evidencing that alteration of the intestinal microbiota of mice has an impact on the infectivity of different enteric viruses by probably affecting similar mechanisms.

Altogether, we have set up a new immunocompetent murine model to study HuNoV replication in vivo. The results obtained show that in this model the microbiota is more prone to have an anti-viral effect, although the human gut microbiota seems to be more permissive to HuNoV infection in mice than the indigenous one. Finally, we showed that an anti-inflammatory environment is probably facilitating HuNoV replication in mice, shedding light on the role of the gut microbiota and its regulation of the immune system in HuNoV replication.

## Data Availability

The raw sequencing fastq files were deposited in the SRA repository (http://www.ncbi.nlm.nih.gov/sra, accessed on 1 July 2021) under Bioproject ID PRJNA706108.

## Supplementary Materials

The following supporting information can be downloaded at: www.mdpi.com/article/10.3390/ijms231810643/s1.
