# Supplementary material for: Replication of Human Norovirus in Mice after Antibiotic-Mediated Intestinal Bacteria Depletion
Source: Int J Mol Sci. 2022 Sep 13;23(18):10643. doi: 10.3390/ijms231810643 (PMC9505278; doi:10.3390/ijms231810643)
Supplement: Supplementary file 1 [file ijms-23-10643-s001.zip › ijms-1862183-supplementary.pptx]

## Slide 1
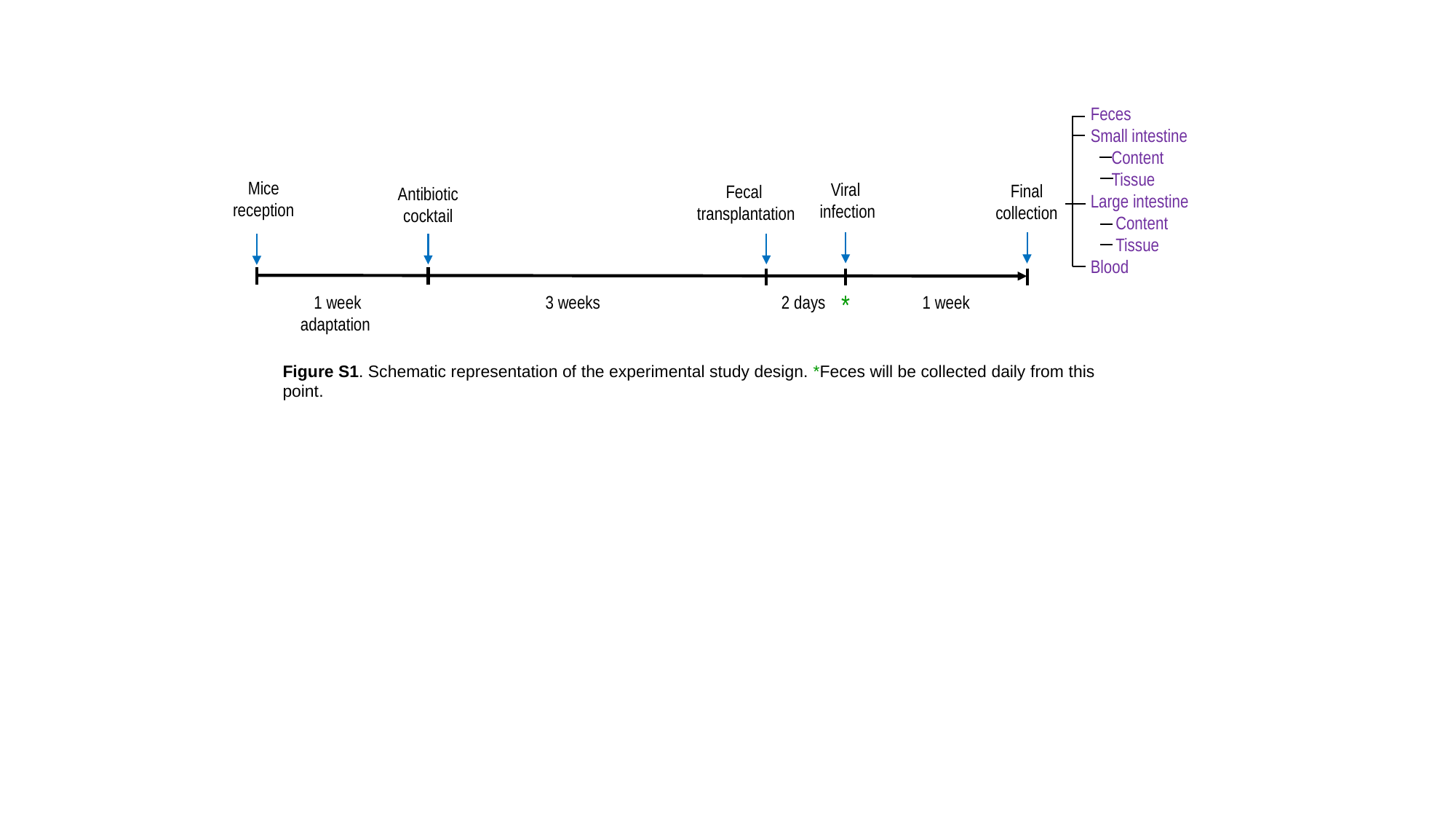

Feces
Small intestine
 Content
 Tissue
Large intestine
 Content
 Tissue
Blood
Mice
reception
Viral
 infection
Final
collection
Fecal
transplantation
Antibiotic
cocktail
*
1 week
adaptation
3 weeks
2 days
1 week
Figure S1. Schematic representation of the experimental study design. *Feces will be collected daily from this point.
